# Biomarkers of cytokine storm as red flags for severe and fatal COVID-19 cases: A living systematic review and meta-analysis

**DOI:** 10.1371/journal.pone.0253894

**Published:** 2021-06-29

**Authors:** Ana Karla G. Melo, Keilla M. Milby, Ana Luiza M. A. Caparroz, Ana Carolina P. N. Pinto, Rodolfo R. P. Santos, Aline P. Rocha, Gilda A. Ferreira, Viviane A. Souza, Lilian D. A. Valadares, Rejane M. R. A. Vieira, Gecilmara S. Pileggi, Virgínia F. M. Trevisani

**Affiliations:** 1 Division of Rheumatology, Department of Clinical Medicine, Hospital Universitário Lauro Wanderley, Universidade Federal da Paraíba, João Pessoa, PB, Brazil; 2 Evidence-Based Health Program, Universidade Federal de São Paulo, São Paulo, SP, Brazil; 3 Department of Pediatric Rheumatology, Hospital de Base, Faculdade de Medicina de São José do Rio Preto, São José do Rio Preto, SP, Brazil; 4 Department of Biological and Health Sciences, Universidade Federal do Amapá, Macapá, AP, Brazil; 5 Fulbright Alumna at University of Pittsburgh, Pittsburgh, PA, United States of America; 6 Department of Data Science, Synova Health, Campinas, SP, Brazil; 7 Department of Lokomotor System, Universidade Federal de Minas Gerais, Belo Horizonte, MG, Brazil; 8 Department of Clinical Medicine, Universidade Federal de Juiz de Fora, Juiz de Fora, MG, Brazil; 9 Department of Rheumatology, Hospital Getúlio Vargas de Pernambuco, Recife, PE, Brazil; 10 Department of Clinical Medicine, Universidade Estadual do Ceará, Fortaleza, CE, Brazil; 11 Department of Clinical Medicine, Universidade de Fortaleza, Fortaleza, CE, Brazil; 12 Department of Rheumatology, Escola Paulista de Medicina, Universidade Federal de São Paulo, São Paulo, SP, Brazil; 13 Department of Rheumatology, Universidade de Santo Amaro, São Paulo, SP, Brazil; The Ohio State University, UNITED STATES

## Abstract

**Objective:**

To describe the laboratory parameters and biomarkers of the cytokine storm syndrome associated with severe and fatal COVID-19 cases.

**Methods:**

A search with standardized descriptors and synonyms was performed on November 28^th^, 2020 of the MEDLINE, EMBASE, Cochrane Central Register of Controlled Trials, ClinicalTrials.gov, LILACS, and IBECS to identify studies of interest. Grey literature searches and snowballing techniques were additionally utilized to identify yet-unpublished works and related citations. Two review authors independently screened the retrieved titles and abstracts, selected eligible studies for inclusion, extracted data from the included studies, and then assessed the risk of bias using the Newcastle-Ottawa Scale. Eligible studies were those including laboratory parameters—including serum interleukin-6 levels—from mild, moderate, or severe COVID-19 cases. Laboratory parameters, such as interleukin-6, ferritin, hematology, C-Reactive Protein, procalcitonin, lactate dehydrogenase, aspartate aminotransferase, creatinine, and D-dimer, were extracted from the studies. Meta-analyses were conducted using the laboratory data to estimate mean differences with associated 95% confidence intervals.

**Data synthesis:**

The database search yielded 9,620 records; 40 studies (containing a total of 9,542 patients) were included in the final analysis. Twenty-one studies (n = 4,313) assessed laboratory data related to severe COVID-19 cases, eighteen studies (n = 4,681) assessed predictors for fatal COVID-19 cases and one study (n = 548) assessed laboratory biomarkers related to severe and fatal COVID-19 cases. Lymphopenia, thrombocytopenia, and elevated levels of interleukin-6, ferritin, D-dimer, aspartate aminotransferase, C-Reactive-Protein, procalcitonin, creatinine, neutrophils and leucocytes were associated with severe and fatal COVID-19 cases.

**Conclusions:**

This review points to interleukin-6, ferritin, leukocytes, neutrophils, lymphocytes, platelets, C-Reactive Protein, procalcitonin, lactate dehydrogenase, aspartate aminotransferase, creatinine, and D-dimer as important biomarkers of cytokine storm syndrome. Elevated levels of interleukin-6 and hyperferritinemia should be considered as red flags of systemic inflammation and poor prognosis in COVID-19.

## Introduction

In December 2019, a new strain of coronavirus, severe acute respiratory syndrome–coronavirus 2 (SARS-CoV-2), emerged in Wuhan, China, and starting in March of 2020, the world was plunged into a pandemic due to the disease (COVID-19) caused by this new coronavirus. As of March 11^th^, COVID-19 had been detected in 221 countries, with 117,799,584 confirmed cases and more than 2,615,018 deaths [1]. The clinical spectrum of this disease ranges from asymptomatic cases to severe pulmonary involvement with respiratory failure, systemic involvement with sepsis, septic shock, and multiple organ failure [2]. Accumulating evidence suggests that a subgroup of patients with severe COVID-19 might have cytokine storm syndrome (CSS). CSS and secondary hemophagocytic lymphohistiocytosis (sHLH) are both associated with cytopenia, hyperferritinemia, and elevated interleukin-6 (IL-6) due to a viral driven hyper-inflammation and amplification of the immune response [3–5].

Elevated serum concentrations of IL-6 and other inflammatory cytokines are hallmarks of CSS and correlate with poor clinical outcomes [3]. Elevated serum C-reactive protein (CRP), a protein whose expression is driven by IL-6, is also a biomarker of severe clinical manifestations of COVID-19. This infection results in monocyte, macrophage, and dendritic cell activation. IL-6 release instigates an amplification cascade that results in cis signaling with TH17 differentiation and trans-signaling in many cell types, such as endothelial cells [3, 5]. The increased systemic cytokine production contributes to the pathophysiology of severe COVID-19 and acute respiratory distress syndrome (ARDS). Recognizing and treating CSS in a timely fashion is of paramount importance, as this information could lead to better outcomes in patients who present this complication as part of their clinical course of COVID-19. We have therefore designed and carried out this living systematic review to guide the evaluation and early recognition of CSS by:

1. Identifying the laboratory parameters correlated with severe and fatal cases of COVID-19;
2. Estimating the role of IL-6 and ferritin as potential biomarkers related to cytokine storm development.

## Methods

### Protocol and registration

This systematic review protocol was registered within the International Prospective Register of Systematic Reviews (PROSPERO), under the code CRD42020190021. We followed the recommendations proposed by the Cochrane Collaboration Handbook [6] and the Preferred Reporting Items for Systematic Reviews and Meta-Analysis (PRISMA) [7].

### Search strategy

We searched MEDLINE, EMBASE, Cochrane Central Register of Controlled Trials (CENTRAL), LILACS, and IBECS using relevant descriptors and synonyms, adapting the search to the specifics of each database (S1 Table). We also searched the Open Grey database, the World Health Organization International Clinical Trials Registry Platform (WHO ICTRP), and ClinicalTrials.gov to identify published, ongoing, and unpublished studies. Finally, we used the technique of snowballing, searching the lists of references of the included studies.

### Eligibility criteria and study selection

Studies were included if they presented laboratory data—including serum IL-6 levels—from mild-to-moderate, severe or critical patients with COVID-19, according to the National Health Commission of China (NHCC) Guidelines for Diagnosis and Management of COVID-19 [8] or World Health Organization Interim Guidance for COVID-19 [9]. Exclusion criteria consisted of studies that assessed pregnant women, pediatric patients, individuals co-infected with other microorganisms, or populations exclusively related to oncological, rheumatological, transplants, or chronic renal disease. All studies published before November 28^th^, 2020 were included, and no language restrictions were implemented for electronic search.

### Data extraction and quality appraisal

We used a predefined form to extract data from included studies. Specifically, we extracted participants and studies characteristics, including: age, gender, diagnostic criteria, and the severity of condition, details on the number of participants screened, randomized, analyzed, excluded, lost to follow-up and dropped out, setting, duration of studies, laboratorial biomarkers, outcome measures, and time points reported. We then assessed the risk of bias inherent to each of the included studies using the modified Newcastle-Ottawa Scale (NOS) [10]. We attempted to contact the authors of the included studies when any study data or other details were missing.

### Summary measures and synthesis of results

Study selection, data extraction, and assessment of the risk of bias were performed independently by two review authors. Disagreements were resolved through discussion or, if required, by consulting a third author. Studies were assessed into two separate groups for analysis: severity group and mortality group. Laboratory parameters, such as IL-6, ferritin, hemoglobin, leukocytes, lymphocytes, neutrophils, platelets, CRP, procalcitonin, lactate dehydrogenase (LDH), aspartate aminotransferase (AST), creatinine, and D-dimer were analyzed. The assays for detecting laboratory tests were similar between studies.

### Statistical analysis

After data was extracted, we used the “meta” package (version 3.4.1) [11] of R software to perform the meta-analysis. For studies presenting continuous data as medians and inter-quartile ranges, we estimated the means and standard deviations according to the method described by Hozo SP et al. [12]. A meta-analysis was performed, with a calculation of mean difference (MD) and 95% confidence interval (95% CI) for each of the laboratory parameters related to patients with confirmed COVID-19 with or without severe disease and non-survivors or survivors. Heterogeneity across the included studies was assessed using a chi-square (χ2) test and the I^2^ statistic. A random effects model was employed to calculate pooled results. A sensitivity analysis was performed to explore the sources of heterogeneity when I^2^ > 50%. We explored the sources of heterogeneity by excluding studies with unclear timepoints of collection and studies that collected blood parameters after seven days of hospital admission. When at least 10 studies were included in a meta-analysis, we assessed the possibility of publication bias using Egger’s test (S2A and S2B Table).

## Results

### Study selection

**Fig 1:**
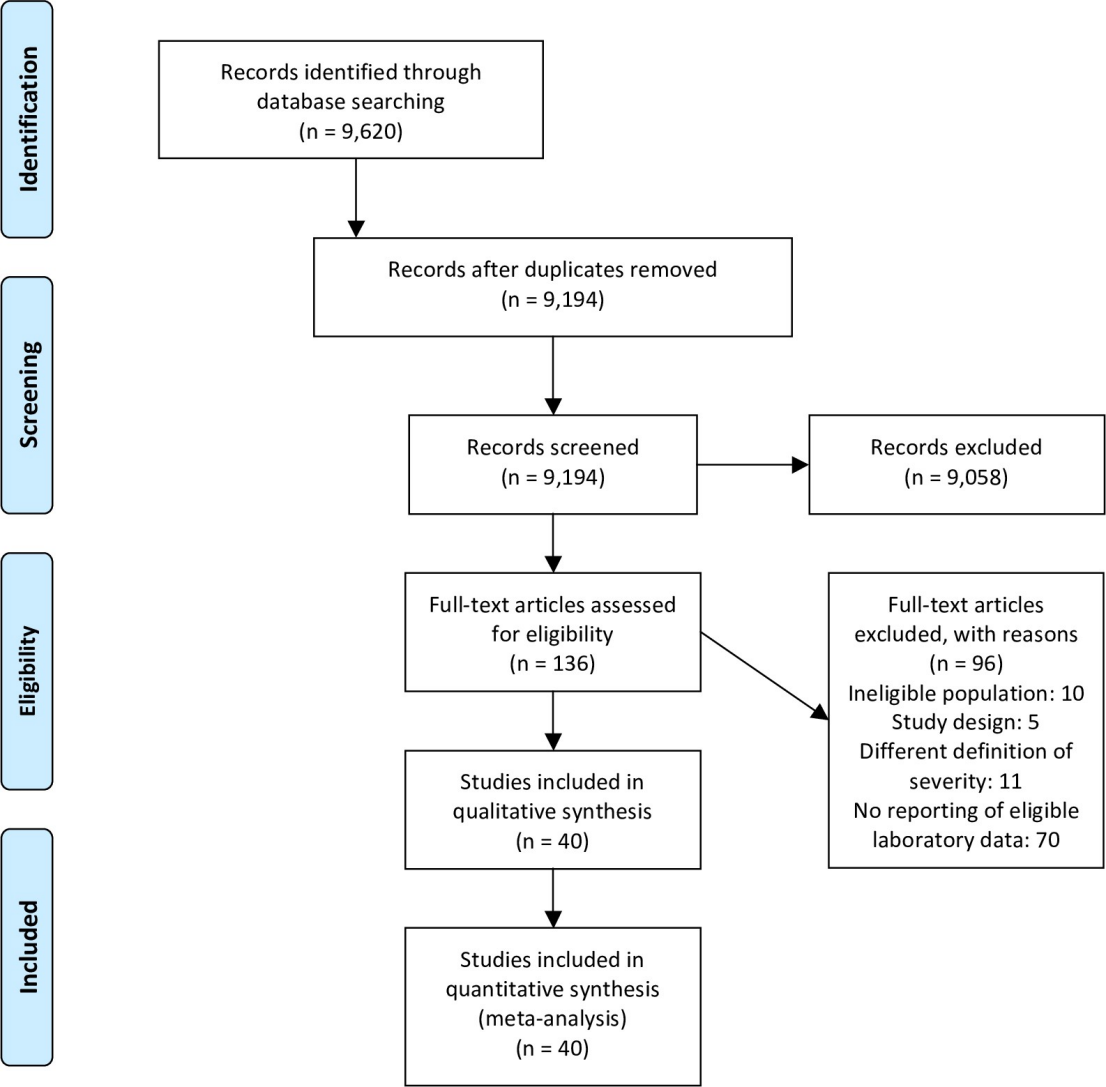
Study selection process flow chart.

The database search yielded 9,620 records. After removing duplicates, 9,194 titles and abstracts were examined. We retrieved 136 full-text articles for further scrutiny; of those, 96 studies were excluded due to: ineligible population(n = 10), study design (n = 5), different definition of severity (n = 11) or not reporting the laboratory data of interest for this meta-analysis (n = 70) (S3 Table). We finally included 40 studies in this review (Fig 1).

### Study characteristics

Thirty-seven eligible studies consisted of retrospective case series and three were prospective cohorts [13–15], conducted in various hospital settings: China (n = 35) [16–31], Israel (n = 1) [32], Brazil (n = 1) [14], Spain (n = 1) [33], Norway (n = 1) [13] and Turkey (n = 1) [34].

The participants (n = 9,542) all received a positive COVID-19 diagnosis through 2019-nCoV RNA detection by SARS-CoV-2 real-time polymerase chain reaction nasopharyngeal swab (RT-PCR) or specific virus IgM and IgG antibodies. Twenty-one studies [16–26] (n = 4,313) assessed laboratory data related to the severity of COVID-19, comparing severe and non-severe cases. Eighteen studies (n = 4,681) [13–15, 27–33, 35–42] evaluated the association between laboratory parameters and mortality in patients with COVID-19 and one study (n = 548) [43] assessed serum biomarkers related to severe and fatal COVID-19 cases. The participants’ baseline and demographic characteristics are presented in Table 1. The descriptive analysis indicates that patients with severe disease were older than non-severe patients, except in two studies [43, 44]. Mortality was also higher in the elderly. Most patients with severe disease and higher mortality were male. Most studies showed a higher prevalence of comorbidities in patients with the severe form of COVID-19.

**Table 1:** Baseline and demographic characteristics of the included studies.

**Assessed outcome: Severity**										
**Study ID**	**Severity criteria**	**Total enrolled patients**	**Severe group**				**Non-Severe group**			
			**No. (%)**	Age*	**Male (%)**	**Comorbidity (%)**	**No. (%)**	Age*	**Male (%)**	**Comorbidity (%)**
Guang Chen, 2020 [16]	Severe vs Moderate cases according to NHCC COVID-19 Guideline (6^th^ Edition) ^a^	21	11 (52.38)	61.0 (56.5–66.0)	10 (90.9)	5 (45.5)	10 (47.62)	52.0 (42.8–56.0)	7 (70.0)	2 (20.0)
Yong Gao, 2020 [18]	Severe vs Mild cases according to WHO Interim Guidance for COVID‐19^c^	43	15 (53.88)	45.20 (± 7.68)	9 (60)	**	28 (46.12)	42.96 (± 14.00)	17 (60.71)	**
Zhongliang Wang, 2020 [19]	Patients with SpO2<90% (Severe) vs Patients with SpO2≥90% (Non-severe) according to NHCC COVID-19 Guidelines (3rd edition)^a^	69	14 (20.29)	70.5 (62.0–77.0)	7 (50)	**	55 (79.71)	37.0 (32.0–51.0)	25 (45)	**
Chuan Qin, 2020 [20]	Severe vs Moderate cases according to NHCC COVID-19 Guidelines (5^th^ Edition)^a^	452	286 (63.27)	61 (51–69)	155 (54.2)	146 (51)	166 (36.73)	53 (41.25–62)	80 (48.2)	55 (33.1)
Chen Lei, 2020 [22]	Severe/Critical vs Mild cases according to NHCC COVID-19 Guidelines (4^th^ Edition)^a^	29	14 (48.28)	NR	NR	**	15 (51.72)	NR	NR	**
Ruirui Wang, 2020 [23]	Critical (Severe or critical cases) vs Non-critical (mild or moderate) cases according to NHCC COVID-19 Guidelines (5^th^ Edition)^a^	125	25 (20)	49.40 (±13.64)	16 (64)	12 (48)	100 (80)	39.47 (±14.84)	55 (55)	22 (22)
Zhe Zhu, 2020 [24]	Severe vs Non-severe cases according to NHCC COVID-19 Guidelines (6^th^ Edition)^a^	127	16 (12.6)	57.50 (±11.70)	9 (56.25)	12 (75)	111 (87.4)	49.95 (±15.52)	73 (65.77)	40 (36.04)
Xiaohua Chen, 2020 [25]	Moderate vs Severe vs Critically ill cases according to NHCC COVID-19 Guidelines (6th Edition)^a^	48	Severe: 10 (20.83) Critically ill: 17 (35.42)	Severe: 63.9 (±15.2) Critically ill: 79.6 (±12.6)	Severe: 9 (90) Critically ill: 15 (88.2)	**	21 (43.75)	52.8 (±14.2)	13 (61.9)	**
Ming Ding, 2020 [26]	Mild vs Severe vs Critical cases according to NHCC COVID-19 Guidelines (7th Edition)^a^	32	Severe: 10 (31.25) Critical: 11 (34.37)	Severe:61.3 (±17.9) Critical: 73.5 (±12.3)	Severe: 5 (50) Critical: 7 (63.63)	Severe: 3 (30) Critical: 4 (36.36)	11 (34.37)	54.9 (±11.3)	1 (9.09)	3 of 5 (60)
Chen LD, 2020 [46]	Mild (without pneumonia) vs Moderate cases with pneumonia (Non-severe) vs Severe cases with pneumonia according to NHCC COVID-19 Guidelines (7th Edition)^a^	106	25 (23.6)	60.68 (± 15.23)	15 (60)	11 (44.0)	Mild: 12 (11.3) Moderate: 69 (65.1)	Mild: 43.92 (± 13.73) Moderate: 51.41 (± 15.77)	Mild: 4 (33.3) Moderate: 34 (49.3)	Mild: 0 (0.0) Moderate: 14 (20.3)
Chen R, 2020 [43]	Mild/Moderate cases vs Severe cases vs Critical cases according to NHCC COVID-19 Guidelines (7th Edition)^a^	548	Severe: 155 (28.3) Critical: 48 (8.8)	Severe: 60.9 (± 13.8) Critical: 61.4 (±13.6)	Severe: 93 (60) Critical: 38 (79.2)	**	345 (62.9)	67.3 (±12.1)	182 (52.75)	**
Chi Y, 2020 [79]	Mild vs Moderate vs Severe cases according to NHCC COVID-19 Guidelines (5th Edition)^a^	66	8 (12.1)	54.0 (± 12.38)	5 (62.5)	4 (50)	Mild: 22 (33.4) Moderate: 36 (54.5)	Mild: 43.32 (± 18.38) Moderate: 40.81 (± 11.8)	Mild: 13 (59.1) Moderate: 19 (53)	Mild: 6 (27) Moderate: 8 (22)
Hu ZJ, 2020 [80]	Severe vs Non-severe cases according to NHCC COVID-19 Guidelines (7^th^ Edition)^a^	76	13 (17.2)	61.5 (57.1–65.9)	8 (61.5)	**	63 (82.8)	48.2 (46.0–50.4)	26 (41.3)	**
Huang Z, 2020 [44]	Moderate (Non-severe) vs Severe vs Critical cases according to NHCC COVID-19 Guidelines (7^th^ Edition)^a^	83	Severe: 29 (35) Critical: 33 (39.7)	Severe: 67 (60–79) Critical: 58 (49–62)	Severe: 16 (55.2) Critical: 26 (78.8)	Severe: 20 (69) Critical: 21 (63.6)	21 (25.3)	68 (57–69)	12 (57.14)	9 (42.86)
Li X, 2020 [47]	Severe (Severe pneumonia/ ARDS) vs Non-severe cases (Mild/ Common pneumonia) according to NHCC COVID-19 Guidelines (7^th^ Edition)^a^ and WHO Interim Guidance for COVID‐19^*c*^	215	56 (26.1)	56.5 (20–72)	36 (64.3)	**	159 (73.9)	44 (32–52)	91 (57.2)	**
Liu D, 2020 [81]	Moderate vs Severe vs Critical cases according to NHCC COVID-19 Guidelines (7^th^ Edition)^a^	2044	Severe: 689 (33.7) Critical: 268 (13.1)	Severe: 64.0 (54.0–71.0) Critical: 69.0 (62.0–77.0)	Severe: 349 (50.65) Critical: 176 (65.67)	Severe: 423/687 (61.57) Critical: 212/266 (79.7)	1087 (53)	59 (46–67)	475 (43.7)	540/1086 (49.72)
Ozsurekci Y, 2020 [34]	Mild vs Moderate vs Severe/ Critical cases according to WHO Interim Guidance for COVID‐19^*c*^	30	11 (36.7)	NR	NR	**	Mild: 4 (13.4) Moderate: 15 (50)	NR	NR	**
Xu X, 2020 [48]	Moderate (non-severe) vs Severe vs Critically ill cases according to NHCC COVID-19 Guidelines (7^th^ Edition)^a^	88	Severe: 32 (36.4) Critically ill: 9 (10.2)	Severe: 59.94 (±13.96)Critically ill: 74.78 (±10.06)	Severe: 8 (25) Critically ill: 7 (77.78)	Severe: 17 (53.13) Critically ill: 7 (77.78)	47 (53.4)	52.49 (±14.62)	21 (44.68)	17 (36.17)
Zeng YL, 2020 [45]	Ordinary (Non-severe) vs Severe vs Critical cases according to NHCC COVID-19 Guidelines (6^th^ Edition)^a^	49	Severe: 16 (32.7) Critical: 5 (10.2)	Severe: 60 (±16) Critical: 68 (±20)	Severe: 8 (50) Critical: 3 (60)	**	28 (57.1)	46 (±19)	15 (53.6)	**
Zeng Z, 2020 [49]	Moderate (Non-severe) vs Severe / Critical cases according to NHCC COVID-19 Guidelines (6^th^ Edition)^a^	317	Severe: 167 (52.68) Critical: 57 (17.98)	Severe: 62.0 (51.0–69.0) Critical: 68.0 (57.0–77.0)	Severe: 90 (53.9) Critical: 31 (54.4)	**	93 (29.34)	59.0 (46.0–68.5)	41 (44.1)	**
Zhao C, 2020 [82]	Mild (Non-severe) vs Severe cases according to WHO Interim Guidance for COVID‐19^*c*^	172	60 (34.8)	70.6 (±11.6)	37 (61.7)	38 (63.3)	112 (65.2)	64 (50–67)	45 (40.2)	57 (50.9)
Zou L, 2020 [83]	Severe vs Non-severe cases according to NHCC COVID-19 Guidelines (3^rd^-6^th^ Edition)^a^	121	52 (42.98)	69.5 (61.5–79.75)	32 (61.5)	52 (88.5)	69 (57.02)	60.0 (52.0–68.0)	34 (49.3)	39 (56.5)
**Assessed outcome: Mortality**										
**Study ID**		**Total enrolled patients**	**Death**				**Survival**			
			**No. (%)**	Age*	**Male (%)**	**Comor**^**b**^**idity (%)**	**No. (%)**	Age*	**Male (%)**	**Comorbidity (%)**
Tao Chen, 2020 [27]		274	113 (41.24)	68 (62–77)	83 (73)	71 (63)	161 (58.76)	51 (37–66)	88 (55)	62 (39)
Lang Wang, 2020 [28]		339	65 (19.18)	76 (70–83)	39 (60)	**	274 (80.82)	68 (64–74)	127 (46.4)	**
Fei Zhou [29], 2020		191	54 (28.2)	69 (63–76)	38 (70)	36 (67)	137 (71.8)	52 (45–58)	81 (59)	55 (40)
Haiying Sun, 2020 [30]		244	121 (49.59)	72 (66–78)	82 (67.8)	**	123 (50.41)	67 (64–72)	51 (41.5)	**
Junli Fan, 2020 [31]		21	4 (19.05)	79.7 (±14.3)	2 (50)	4 (100)	17 (80.95)	61.5 (±9.5)	9 (53.9)	9 (53.9)
Laguna-Goya R, 2020 [15]		501	36 (7.18)	65 (57–72)	25 (69.4)	**	465 (92.82)	52 (44–58)	292 (62.9)	**
Chen F, 2020 [36]		660	82 (12.42)	71 (63‐83)	58 (70.7)	64 (78)	578 (87.58)	54 (37‐66)	237 (41)	262 (45.3)
Chen H, 2020 [37]		172	87 (50.58)	71 (61–78)	50 (57.47)	**	85 (49.42)	53 (43–65)	44 (51.76)	**
Gadotti AC, 2020 [14]		56	18 (32.14)	66 (56–77)	16 (88.8)	**	38 (67.86)	56 (43–72)	23 (60.52)	**
Chen R, 2020 [43]		548	103 (18.8)	66.9 (±12.1)	69 (67)	**	445 (81.2)	53.5 (±13.9)	244 (54.83)	**
Guirao JJ, 2020 [33]		50	14 (28)	69.00 (± 3.09)	11 (78.57)	**	36 (72)	61.36 (± 1.72)	30 (83.3)	**
Ke C, 2020 [35]		194	46 (23.7)	69.76 (±10)	32 (69.57)	**	148	60.30 (±14.56)	83 (56.08)	**
Li C, 2020 [38]		476	183 (38.4)	71 (62–79)	121 (66.12)	123 (67.2)	293 (61.6)	68 (59–77)	161 (54.95)	196 (66.9)
Luo M, 2020 [39]		1018	201 (19.7)	69 (62–78)	133 (66.2)	**	817 (80.3)	57 (46–66)	388 (47.5)	**
Mandel M, 2020 [32]		71	12 (16.9)	NR	NR	NR	59 (83.1)	NR	NR	NR
Wang ZH, 2020 [40]		59	41 (69.5)	70.2 (±9.0)	26 (63.4)	**	18 (30.5)	61 (±13.5)	12 (66.7)	**
Zhang B, 2020 [41]		98	36 (36.7)	70.5 (±1.7)	23 (64)	**	62 (63.3)	60.0 (±1.9)	35 (56.5)	**
Zhang L, 2020 [42]		134	101 (75.37)	65.46 (±9.74)	64 (63.4)	**	33 (24.63)	46.45 (±11.09)	23 (69.70)	**
Myhre PL, 2020 [13]		123	8 (6.5)	NR	NR	**	115 (93.5)	NR	NR	**

SpO2 = Blood oxygen saturation level, NR = Not reported.

a National Health Commission of China Guidelines for Diagnosis and Management of COVID-19 [84].

c World Health Organization Interim Guidance for COVID‐19 [85].

* Values expressed in Median (interquartile range) or Mean ± SD (standard deviation).

** study does not describe the exact prevalence of overall comorbidities in each group.

### Risk of bias assessment

The studies’ risk of bias, as measured on the Newcastle-Ottawa Scale, as suggested by Bazerbachi 2018 [10], ranged from 2/5 [19, 45] to 5/5 [15, 20, 23, 24, 29, 37, 43, 46–49], with higher scores indicating lower risk of bias (Table 2). All evaluated studies succeeded in excluding cases that were not pertinent to the research question. The domain that revealed the highest frequency of negative assessment was the one related to the representativeness of the exposed group. Detailed information on each of the domains is provided in Table 2.

**Table 2:** Risk of bias assessment—Modified Newcastle-Ottawa Scale [10].

Study ID, Year	Selection			Outcome		NOS
	Did the sample represent the whole cases of interest?	Was the diagnosis correctly made	Was the other important diagnosis excluded?	Were all the important data cited in the report	Was the outcome correctly ascertained	
Guang Chen [16], 2020	-	+	+	+	+	4/5
Yong Gao [18], 2020	-	+	+	+	-	3/5
Zhongliang Wang [19], 2020	-	+	+	-	-	2/5
Chuan Qin [20], 2020	+	+	+	+	+	5/5
Chen Lei [22], 2020	-	+	+	+	+	4/5
Ruirui Wang [23], 2020	+	+	+	+	+	5/5
Zhe Zhu [24], 2020	+	+	+	+	+	5/5
Xiaohua Chen [25], 2020	-	+	+	+	-	3/5
Ming Ding [26], 2020	-	+	+	+	+	4/5
Tao Chen [27], 2020	+	-	+	+	+	4/5
Lang Wang [28], 2020	+	-	+	+	+	4/5
Fei Zhou [29], 2020	+	+	+	+	+	5/5
Haiying Sun [30], 2020	+	+	+	-	-	3/5
Junli Fan [31], 2020	-	-	+	+	+	3/5
Chen LD, 2020 [46]	+	+	+	+	+	5/5
Chen R, 2020 [43]	+	+	+	+	+	5/5
Chi Y, 2020 [79]	-	+	+	+	+	4/5
Guirao JJ, 2020 [33]	-	+	+	-	+	3/5
Hu ZJ, 2020 [80]	-	+	+	+	-	3/5
Huang Z, 2020 [44]	-	+	+	+	-	3/5
Li X, 2020 [47]	+	+	+	+	+	5/5
Liu D, 2020 [81]	+	+	+	+	-	4/5
Ozsurekci Y, 2020 [34]	-	+	+	+	+	4/5
Xu X, 2020 [48]	+	+	+	+	+	5/5
Zeng YL, 2020 [45]	-	+	+	-	-	2/5
Zeng Z, 2020 [49]	+	+	+	+	+	5/5
Zhao C, 2020 [82]	+	+	+	+	-	4/5
Zou L, 2020 [83]	+	+	+	+	-	4/5
Myhre PL, 2020 [13]	-	+	+	+	+	4/5
Laguna-Goya R, 2020 [15]	+	+	+	+	+	5/5
Chen F, 2020 [36]	+	-	+	+	+	4/5
Chen H, 2020 [37]	+	+	+	+	+	5/5
Gadotti AC, 2020 [14]	-	+	+	+	-	3/5
Ke C, 2020 [35]	-	-	+	+	+	3/5
Li C, 2020 [38]	+	+	+	-	+	4/5
Luo M, 2020 [39]	+	-	+	+	+	4/5
Mandel M, 2020 [32]	-	+	+	-	+	3/5
Wang ZH, 2020 [40]	-	+	+	+	-	3/5
Zhang L, 2020 [42]	-	+	+	+	+	4/5
Zhang B, 2020 [41]	-	+	+	+	-	3/5

NOS = Newcastle-Ottawa Scale

### Synthesis of results

Laboratory parameters in severe versus non-severe patients and non-surviving versus surviving patients with COVID-19 are described in Table 3. Lymphopenia, thrombocytopenia and higher levels of IL-6, ferritin, D-dimer, aspartate aminotransferase, lactate dehydrogenase, CRP, procalcitonin, creatinine, neutrophils and leukocytes were associated with severe and fatal cases of COVID-19. Abnormalities of hemoglobin were not associated with disease severity or increased mortality. We explored the sources of heterogeneity by excluding studies with unclear timepoints of collection and studies that collected blood parameters after 7 days of hospital admission. However, no difference in the statistical heterogeneity across studies could be detected in this sensitivity analysis, except for d-dimer [MD 5.94; CI 5.16 to 6.73; N = 2,459; I^2^ = 0.19] and creatinine [MD 19.44; CI 16.79 to 22.10; N = 1,689; I^2^ = 0.49] in the mortality group.

**Table 3:** Results of meta-analysis comparing laboratory parameters in COVID-19 patients.

	SEVERE versus NON-SEVERE					FATAL versus NON-FATAL				
Parameter	Number of studies	MD	Random; 95% CI	I^2^	Number of participants	Number of studies	MD	Random; 95% CI	I^2^	Number of participants
Haemoglobin	9	-0.04	-0.37; 0.29	0.84	3,523	7	-0.10	-0.45; 0.25	0.91	2,378
White Blood Cell (WBC)	17	1.63	0.98; 2.27	0.97	4,655	13	3.71	3.04; 4.38	0.94	2,990
Lymphocytes	19	-0.4	-0.48; -0.32	0.97	4,733	13	-0.43	-0.45; -0.40	0.59	3,435
Neutrophils	13	2.12	1.18; 3.07	0.98	2,268	8	3.99	2.85; 5.13	0.96	2,613
Platelets	11	-24.22	-36.26; -12.18	0.93	3,726	9	-46.68	-49.99; -43.37	0	2,571
C-Reactive Protein	14	53.54	39.79; 67.29	0.97	4,138	15	58.48	43.35; 73.61	0.99	3,755
Procalcitonin	11	0.08	0.03; 0.14	0.99	3,480	11	0.24	0.13; 0.36	0.96	2,845
LDH	10	153.58	87.09; 220.08	0.95	3,050	10	230.99	192.29; 269.70	0.96	2,622
Creatinine	10	8.07	4.28; 11.87	0.85	3,036	9	17.93	11.89; 23.98	0.91	2,174
AST	8	8.29	0.88; 15.7	0.90	649	8	16.81	8.08; 25.54	0.88	1,843
D-Dimer	9	2.15	0.68; 3.63	0.99	3,181	14	4.64	3.03; 6.24	0.97	3,965
Ferritin	6	654.4	383.48; 925.33	0.96	3,470	9	853.43	601.20; 1105.67	0.94	2,088
IL-6	22	28.93	18.18; 39.69	0.99	4,861	19	70.82	45.24; 96.41	0.96	5,229

MD = mean difference, CI = confidence interval, LDH = lactate dehydrogenase, AST = aspartate aminotransferase, IL-6 = interleukin-6

### Meta-analysis of laboratory data

**Fig 2:**
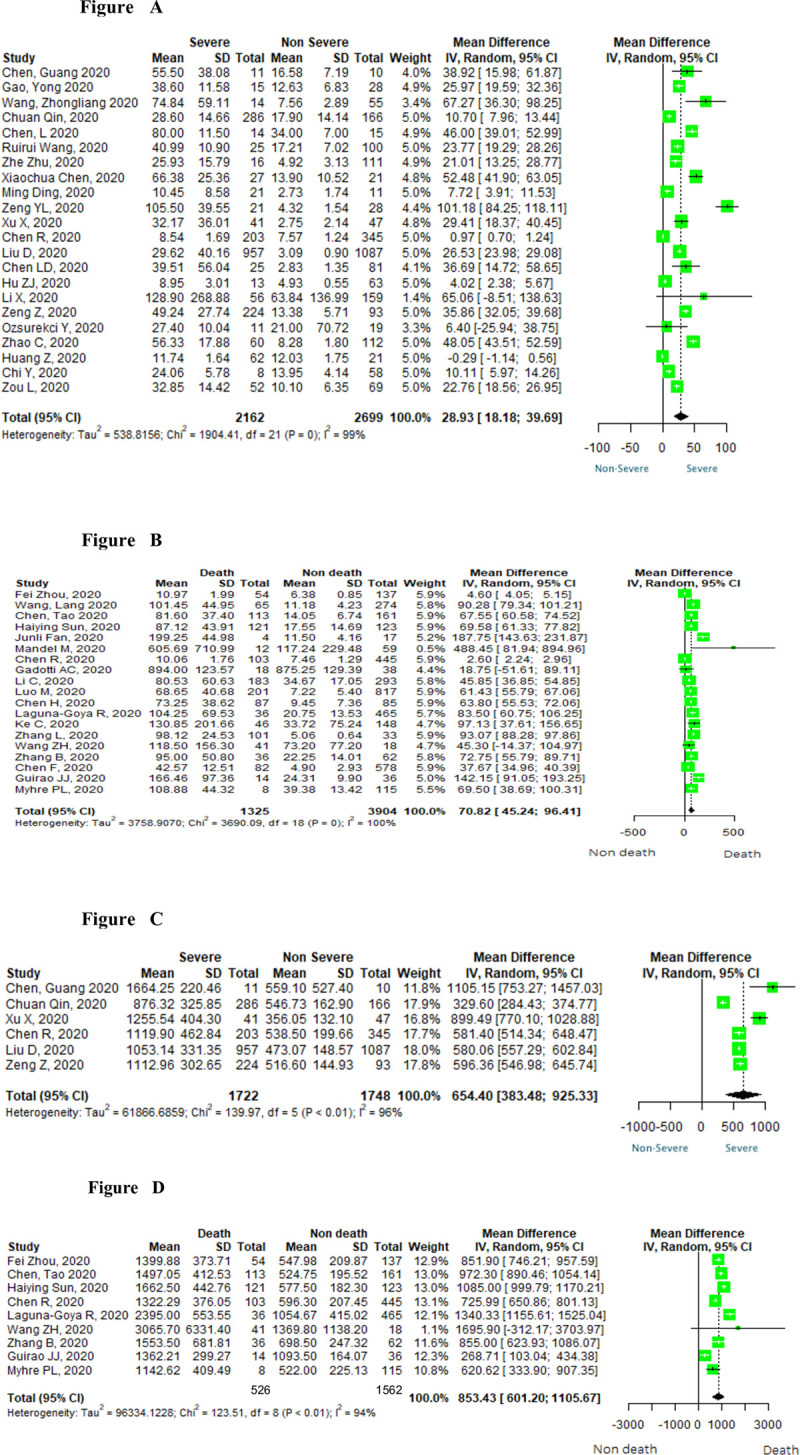
Forest plots of interleukin-6 (2A and 2B) and of ferritin (2C and 2D) in severe and fatal cases of COVID-19.

Nineteen studies assessed fatal and non-fatal groups of patients with COVID-19 and reported IL-6. Pooled results from these studies showed that the patients who progressed to death had higher levels of IL-6 in comparison with those who survived [MD 70.82; 95% CI 45.24 to 96.41; N = 5,229] (Fig 2A). Pooled results from twenty-two studies of both severe and non-severe cases revealed higher levels of Il-6 in patients with severe COVID-19 in comparison to the group of mild COVID-19 cases [MD 28.99; 95% CI 18.24 to 39.75; N = 4,861] (Fig 2B). The pooled analysis of nine studies showed that patients with COVID-19 who did not survive also had higher levels of ferritin [MD 853.43; 95% CI 601.20 to 1105.67; N = 2,088] (Fig 2C). Ferritin levels were reported in six studies [16, 20, 21], with the meta-analysis of severe and non-severe patients showing that patients with severe COVID-19 had higher levels of ferritin [MD 654.40; 95% CI 383.48 to 925.33; N = 3,470] (Fig 2D). Sensitivity analysis was conducted on the IL-6 parameters. Five studies were found to be outliers in the mortality group [14, 29, 32, 33, 43], and three studies in the severity group [20–22]. There was no change in the direction of the effect of the meta-analysis after their exclusion from this calculation [MD 75.15; 95% CI 56.04 to 94.26; N = 4,313] and [MD 33.01; 95% CI 21.79 to 44.24; N = 4,200], respectively. We used the Egger’s test to assess publication bias, and the test suggested the presence of publication bias in the meta-analysis on the association between IL-6 and severity (p<0.001). No publication bias was detected in any other analyses (S2A and S2B Table).

## Discussion

A cytokine profile resembling secondary hemophagocytic lymphohistiocytosis is associated with severe COVID-19 (2–4, [50]. In this living systematic review with meta-analysis, a clear pattern of hematological, biochemical, inflammatory, and immune biomarker abnormalities could be found between patients with or without severe disease. These differences could allow for the identification of predictors of severe and fatal cases. We found that lymphopenia, thrombocytopenia and high levels of ferritin, D-dimer, aspartate aminotransferase, lactate dehydrogenase, C-Reactive Protein, neutrophils, procalcitonin and creatinine are good indicators of both severe and fatal cases of COVID-19 during the first days from illness onset, as is interleukin-6, the main cytokine involved in the CSS.

As high inflammation is a primary cause of pathology in COVID-19, targeted anti-inflammatory treatments are being evaluated to reduce inflammation-induced damage to the respiratory tract and to mitigate the cytokine storm [51, 52]. Based on observational studies [53, 54], COVID-19 progression has now been divided into three clinical phases: the viremia phase, the acute phase (pneumonia), and the recovery phase. During the progression of the phases, as mentioned by Lin L et al. [54], cells T and B reduce, while inflammatory cytokines and D-Dimer increase in severe patients. This progression has led some authors to suggest that anti-inflammatory treatment should be started in the acute phase to inhibit the inflammatory storms [54, 55]. One of the central challenges to defining initial treatment for patients with COVID-19 is the early identification of those individuals who will evolve into more severe forms of the disease and of those who require specific interventions or treatments.

Several laboratory differences were observed between severe and non-severe disease. Yuan X et al. [56] demonstrated that patients with severe or critical COVID-19 had decreased red blood cells compared to non-severe patients. Anemia occurs in approximately 80% of patients with sHLH, plays an important role in its pathophysiology, and can be a cause of poor prognosis on these patients [57]. Nonetheless, our meta-analysis failed to find an association of low hemoglobin levels with severity or mortality rates among patients with COVID-19-related cytokine storms.

The increased production (myelopoiesis) and mobilization of monocyte and neutrophil populations from the bone marrow is a response to many acute infections—including viral infections—and cytokines [58]. These cells are typically considered proinflammatory and are recruited to the sites of inflammation where they can respond to pathogen-associated molecular patterns (PAMPs) and damage-associated molecular patterns (DAMPs) by producing interleukin-1, interleukin-6, interleukin-12, and tumor necrosis factor (TNF) [59]. Our data indicate that the increase in white blood cells is associated with elevated neutrophils, and this finding may signify clinical deterioration and increased risk of a poor outcome.

Despite an increase in white blood cell count, we found that lymphocytes were significantly decreased in patients with severe COVID-19. This result is consistent with those reported by Chen G et al. [16], who demonstrated that the total lymphocyte count, and specifically CD4+ T cells and CD8+ T cells, were slightly lower in moderate cases and significantly decreased in severe COVID-19. This study concluded that the cytokine storm is associated with COVID-19 severity, likely through increased pulmonary pathology, T cell depletion, and CD4+ T cell dysfunction.

Our meta-analysis also found an association between lymphopenia and higher mortality. Wang F et al. [60] assessed the levels of peripheral lymphocyte subsets by flow cytometry in 60 hospitalized patients with COVID-19 before and after treatment and their association with clinical characteristics and treatment efficacy. The authors reported that total lymphocytes, CD4+ T cells, CD8+ T cells, B cells, and natural killer (NK) cells were decreased among patients with COVID-19, more so in severe cases than in mild cases. In their analysis, the subsets of lymphocytes showed a significant association with inflammatory status in COVID-19, especially CD8+ T cells and CD4+/CD8+ ratio.

Thrombocytopenia is a recurrent complication among patients requiring intensive care, regardless of the cause of hospitalization, and represents a marker of poor prognosis [61]. In COVID-19, it is speculated that SARS-CoV-2 may inhibit hematopoiesis in the bone marrow, decreasing platelet production [62]. This phenomenon, combined with sepsis-induced disseminated intravascular coagulation, may justify the occurrence of thrombocytopenia in a subgroup of patients with COVID-19 [63]. Our meta-analysis demonstrated that thrombocytopenia could be a predictor of severity and mortality in patients with COVID-19.

Many inflammatory factors can cause systemic damage and multi-organ failure. High serum levels of CRP, AST, LDH, and ferritin in patients with COVID-19 may indicate that liver dysfunctions have been involved, and target treatments should be taken in time to prevent irreversible organ damage and increased risk of mortality [64]. Liver damage in these patients might be directly caused by the viral infection of liver cells; indeed, pathological studies [65, 66] have confirmed the presence of the SARS-CoV in liver tissues.

Procalcitonin levels are generally undetectable in healthy individuals and remain unchanged or moderately increase in those with virus infection or systemic inflammatory diseases. However, its levels increase significantly in cases of generalized infection, mainly bacterial or fungal [67]. In this systematic review, procalcitonin levels increased significantly in non-survivors but only showed a small effect size in studies comparing severe versus non-severe patients. Considering that procalcitonin is a good predictor of bacterial infection, the non-survivors possibly progressed to sepsis, an often-fatal complication in patients with COVID-19. In line with this hypothesis, in the meta-analysis published by Lippi G and Plebani M [68], a significant increase in procalcitonin levels was associated with bacterial co-infection, progression to severe forms of COVID-19, and death.

The activation of the coagulation process is a fundamental mechanism of the etiopathogenesis of SARS-CoV-2 infections and often leads to poor outcomes. Ultimately fatal cases often evolve from life-threatening disseminated intravascular coagulation requiring prompt intervention [69]. Recent clinical experiences with anticoagulants suggest that these substances are associated with a lower risk of thromboembolic disease and severe ischemic manifestations in some patients. Thus, it is imperative to identify patients at high risk for early anticoagulation in COVID-19. Tang N et al. [70] reported that the non-surviving patients with COVID-19 had higher fibrin-related markers (D-dimer and fibrin degradation product) at admission compared with survivors. The same authors also reported that administering low molecular weight heparin in severe patients infected with SARS-CoV-2 with elevated D-dimer or those with sepsis-induced disseminated intravascular coagulation was significantly associated with improved survival. In the present systematic review, increased levels of D-dimer at hospital admission could be a good predictor of severe and fatal cases of COVID-19. Another meta-analysis showed a similar result of the discriminatory ability of D-dimer in patients with and without severe forms of COVID-19 but did not provide data on mortality [71].

Several studies suggested that markedly elevated ferritin in hospitalized patients is often associated with hemophagocytic lymphohistiocytosis syndrome, but cannot be considered as a specific marker [72–74]. Ferritin is an acute protein that increases in response to a broad spectrum of inflammatory states, including infections, malignancy, iron overload, and liver or kidney disease [75, 76]. In our meta-analysis, the findings clarify the role of ferritin in the assessment of the systemic hyper-inflammation and can explain the relationship between hyperferritinemia and high levels of IL-6 among adult patients with COVID-19. Regarding immunological biomarkers, we believe that both parameters can be used as red flags for severe and fatal cases of COVID-19 infection at hospital admission. Additionally, ferritin and C-reactive protein appear to be screening tools for the early diagnosis of a systemic inflammatory response syndrome in patients with a severe form of COVID-19 (denominated CSS), with lower cost and wider availability in frontline clinical practice than IL-6.

Despite these rigorous efforts to guide clinicians in diagnosing CSS, discriminating this pathology from other conditions, particularly sepsis or disseminated intravascular coagulation, remains challenging due to the large degree of overlap in clinical presentation [77]. Therefore, biomarkers that identify which patients will evolve with the severe forms of COVID-19 have substantial clinical applicability. Our review demonstrated that older age, male gender, and comorbidities represent potential risk factors for severe and fatal cases, as has already been reported in the scientific literature. This review also confirms that certain laboratory tests that are part of routine care have also been reliably associated with severe and fatal cases of COVID-19, and including serum IL-6 could be relevant for prognosis but could also improve therapeutic decision making.

### Limitations

The main limitations of this review stem from the nature of the included studies; all included studies were observational as they were published with the inherent urgency of a global pandemic. Although these are study designs with a lower level of evidence, the Newcastle-Ottawa Scale assessment of the risk of bias of the included studies did not show any salient risks. Another chief limitation lies in the fact that most patients are of Asian origin, which may compromise the external validity of this meta-analysis.

Assessing the statistical heterogeneity of the included studies is an essential component of interpreting the results of any meta-analysis. In the present study, the meta-analysis of most outcomes showed considerable heterogeneity, requiring a more detailed examination of the studies in order to determine possible causes for this issue. We attempted to explore sources of statistical heterogeneity by thoroughly examining clinical and methodological heterogeneity across studies and by performing sensitivity analysis. While we explored the sources of heterogeneity in the meta-analyses, we could not find differences that could explain the sources of heterogeneity across studies.

As the clinical course of patients with COVID-19 may widely vary across individuals, unexplored clinical parameters may explain this heterogeneity. Different definitions of the term “severity” adopted by each author, some variables such as the time taken to collect laboratory tests or the number of the days from symptoms onset of COVID-19 to hospital admission may also have notably contributed to this circumstance. Discrepancies in reporting and detailing patients’ comorbidities in each study may also have reinforced heterogeneity. Although considerable, the heterogeneity does not compromise the robustness of this meta-analysis, as it did not interfere with the direction and the magnitude of effects. As this study is underpowered to investigate the underlying mechanism of these inflammatory markers with the severity of COVID-19, further studies aiming at analyzing pathophysiological mechanisms of COVID-19 are needed.

We also examined evidence of publication bias in our meta-analyses, and the presence of non-reporting bias was suggested in the meta-analysis on the association of IL-6 with severity. As we found important heterogeneity in this meta-analysis and tests for adjusting for funnel plot asymmetry such as the trim and fill method are known to perform poorly when there is large between-study heterogeneity [78], we did not perform trim and fill test. Of note, small-study effects may be due to reasons other than publication bias, such as heterogeneity [6]. It is likely that asymmetry in this meta-analysis is also due to heterogeneity. We believe that IL-6 acts as a major pro-inflammatory mediator for the acute severe systemic inflammatory response, leading to a wide range of local and systemic changes, leucocytes recruitment and activation and hemodynamic effects. Thus, unexplored pathophysiological mechanisms may justify this heterogeneity and those results.

Systematic reviews and meta-analysis of prospective studies, with standardized COVID-19 severity criterion in a more global population sample, could be the key to better understanding the cytokine storm influences in patients infected with SARS-CoV-2 and their outcomes. We believe that the results of our review and meta-analysis including a relevant number of patients, especially those related to IL-6 and ferritin, can be extrapolated to the real world, supporting clinical practice in coping with COVID-19 and may help decision-making processes. As this is a living review, we are committed to keeping it updated as new evidence is published.

## Conclusions

This review points to lymphopenia, thrombocytopenia, neutrophilia, leukocytosis as well as increased levels of IL-6, ferritin, D-dimer, aspartate aminotransferase, lactate dehydrogenase, procalcitonin, creatinine and CRP as indicators of severe and fatal cases of COVID-19. Ferritin and IL-6 are significant biomarkers of CSS, and increases in levels of either could be considered red flags of systemic inflammation and poor prognosis in COVID-19, especially in the oldest patients and those with comorbidities.

## Supporting information

S1 Checklist (DOC)

S1 Table Search descriptors. (DOCX)

S2 Table. A. Assessment of publication bias using Egger’s test on the meta-analyses including 10 or more studies on association between laboratory parameters and mortality in patients with COVID-19. B. Assessment of publication bias using Egger’s test on the meta-analyses including 10 or more studies on association between laboratory parameters and severity in patients with COVID-19 (DOCX)

S3 Table Excluded studies. (DOCX)
