# Supplementary material for: Biomarkers of cytokine storm as red flags for severe and fatal COVID-19 cases: A living systematic review and meta-analysis
Source: PLoS One. 2021 Jun 29;16(6):e0253894. doi: 10.1371/journal.pone.0253894 (PMC8241122; doi:10.1371/journal.pone.0253894)
Supplement: S1 Table — (DOCX) [file pone.0253894.s002.docx]

**S1 Table. Search descriptors**

| **Medline via Pubmed**  #1 (("Coronavirus"[Mesh]) OR ("Coronaviridae"[Mesh]) OR ("Coronavirus Infections"[Mesh]) OR coronavirinae OR ("COVID-19" [Supplementary Concept]) OR COVID OR ("severe acute respiratory syndrome coronavirus 2"[Supplementary Concept]) OR SARS-CoV-2 OR ("betacoronavirus"[MeSH Terms]) OR Coronaviruses OR 2019-nCoV OR nCoV OR COVID19 OR (Corona virus)  #2 (("Macrophage Activation Syndrome"[Mesh]) OR ("Lymphohistiocytosis, Hemophagocytic"[Mesh]) OR (Erythrophagocytic Lymphohistiocytoses) OR (Erythrophagocytic Lymphohistiocytosis) OR (Hemophagocytic Histiocytoses) OR (Hemophagocytic Histiocytosis) OR (Hemophagocytic Syndrome) OR (hemophagocytic lymphohistiocytosis) OR (cytokine storm) OR hypercytokinemia OR (critically ill) OR severe OR distress OR (acute respiratory distress syndrome) OR (organ failure) OR ("Cytokine Release Syndrome"[Mesh]) OR ("Systemic Inflammatory Response Syndrome"[Mesh]) OR (Sepsis Syndrome) OR ("Inflammation"[Mesh]))  #3  (clinical features) OR (red flags) OR (warning signs) OR ("Symptom Flare Up"[Mesh]) OR symptomatology OR exarcerbation OR worsening OR (disease marker)  #4. #1 AND #2 AND #3 |
| --- |

| **EMBASE**  #1 'coronavirinae'/exp OR coronavirinae OR 'coronavirus'/exp OR coronavirus OR 'corona virus' OR 'coronaviridae'/exp OR coronaviridae OR 'coronavirus infection'/exp OR 'coronavirus infection' OR 'SARS coronavirus'/exp OR 'SARS coronavirus' OR 'covid-19' OR covid OR 'sars-cov-2' OR coronaviruses OR 'coronavirus infection' OR 'coronavirus disease 2019'/exp OR 'sars-related coronavirus'/exp OR 'Betacoronavirus'/exp OR beta coronavirus    #2 'macrophage activation syndrome'/exp OR 'macrophage activation syndrome' OR 'erythrophagocytic lymphohistiocytosis' OR 'haemophagocytic lymphohistiocytosis' OR 'haemophagocytic syndrome' OR 'haemophagocytic syndromes' OR 'hemophagocytic lymphohistiocytosis' OR 'hemophagocytic syndromes' OR 'lymphohistiocytosis, erythrophagocytic' OR 'lymphohistiocytosis, haemophagocytic' OR 'lymphohistiocytosis, hemophagocytic' OR 'cytokine storm'/exp OR 'cytokine storm' OR cytokine OR hypercytokinaemia OR hypercytokinemia OR 'cytokine release'/exp OR 'cytokine secretion' OR 'release, cytokine' OR 'secretion, cytokine' OR 'clinical feature'/exp OR 'clinical aspect' OR 'clinical features' OR 'disease severity assessment'/exp OR 'disease severity scale' OR 'disease severity score' OR 'severity of disease assessment' OR 'severity of disease scale' OR 'severity of disease score' OR 'symptomatology'/exp OR 'disease marker'/exp OR 'symptom'/exp OR 'disease exacerbation'/exp OR 'aggravation, disease' OR 'disease aggravation' OR 'disease flare' OR 'disease progression' OR 'exacerbation, disease' OR 'inflammation'/exp OR 'systemic inflammatory response syndrome'/exp OR 'sepsis'/exp  #3 #1 AND #2  #4 #3 AND (2019:py OR 2020:py)  #5 #4 AND [embase]/lim NOT ([embase]/lim AND [medline]/lim) |
| --- |

| **LILACS**  MH:Coronavirus OR MH:Betacoronavirus OR MH:B04.820.504.540.150$ OR Coronavirus OR "COVID-19" OR COVID OR SARS-CoV-2 OR Coronaviruses OR MH:Coronaviridae OR MH:B04.820.504.540$ OR coronavirinae  AND  MH:"Macrophage Activation Syndrome" OR MH:C20.683.515.800$ OR (Síndrome de Activación Macrofágica) OR (Síndrome de Ativação Macrofágica) OR (Síndrome da Ativação do Macrófago) OR (Síndrome de Ativação de Macrófagos) MH:"Lymphohistiocytosis, Hemophagocytic" OR (Linfohistiocitosis Hemofagocítica) OR (Linfo-Histiocitose Hemofagocítica) OR (Linfo-Histiocitose Hemofagocítica) OR (Síndrome Hemofagocítica) OR (Síndromes Hemofagocíticas) OR (Erythrophagocytic Lymphohistiocytoses) OR (Erythrophagocytic Lymphohistiocytosis) OR (Hemophagocytic Histiocytoses) OR (Hemophagocytic Histiocytosis) OR (Hemophagocytic Syndrome) OR (hemophagocytic lymphohistiocytosis) OR "cytokine storm" OR hypercytokinemia OR cytokine OR MH:C15.604.250.410.575$ OR (cytokine release syndrome) OR MH:"Systemic Inflammatory Response Syndrome" OR MH:C23.550.470.790$ OR MH:C23.550.835.900$ OR (Sepsis Syndrome) OR MH:Inflammation OR MH:C23.550.470$  AND  (clinical features) OR (red flags) OR MH:"Symptom Flare Up" OR MH:C23.550.291.937.500$ OR symptomatology OR exarcerbation OR worsening OR (disease marker) |
| --- |

| **Cochrane**  #1 MeSH descriptor: [Coronavirus] explode all trees  #2 MeSH descriptor: [Coronaviridae] explode all trees  #3 MeSH descriptor: [Betacoronavirus] explode all trees  #4 "COVID-19" OR (COVID) OR (Coronavirus) OR (SARS-CoV-2) OR (Coronaviruses)  #5 MeSH descriptor: [Macrophage Activation Syndrome] explode all trees  #6 MeSH descriptor: [Lymphohistiocytosis, Hemophagocytic] explode all trees  #7 MeSH descriptor: [Inflammation] explode all trees  #8 (Erythrophagocytic Lymphohistiocytoses) OR (Erythrophagocytic Lymphohistiocytosis) OR (Hemophagocytic Histiocytoses) OR (Hemophagocytic Histiocytosis) OR (Hemophagocytic Syndrome) OR (hemophagocytic lymphohistiocytosis) OR (critically ill) OR severe OR distress OR (acute respiratory distress syndrome) OR (organ failure) OR (cytokine storm) OR hypercytokinemia OR cytokine OR (Sepsis Syndrome)  #9 #1 OR #2 OR #3 OR #4  #10 #5 OR #6 OR #7 OR #8  #11 #9 AND #10 |
| --- |

| **OpenGrey**  COVID-19 OR coronaviridae OR COVID OR Betacoronavirus OR Coronavirus OR SARS-CoV-2 OR Coronaviruses |
| --- |

| **ClinicalTrials.Gov**  Condition: Coronavirus OR Coronaviridae OR COVID-19 OR COVID OR "severe acute respiratory syndrome coronavirus 2" OR SARS-CoV-2 OR "betacoronavirus" OR Coronaviruses |
| --- |
