# Supplementary material for: Biomarkers of cytokine storm as red flags for severe and fatal COVID-19 cases: A living systematic review and meta-analysis
Source: PLoS One. 2021 Jun 29;16(6):e0253894. doi: 10.1371/journal.pone.0253894 (PMC8241122; doi:10.1371/journal.pone.0253894)
Supplement: S2 Table. A. Assessment of publication bias using Egger’s test on the meta-analyses including 10 or more studies on association between laboratory parameters and mortality in patients with COVID-19. B. Assessment of publication bias using Egger’s test on the meta-analyses including 10 or more studie — (DOCX) [file pone.0253894.s003.docx]

**S2A Table. Assessment of publication bias using Egger’s test on the meta-analyses including 10 or more studies on association between laboratory parameters and mortality in patients with COVID-19**

| **Laboratory parameter** | **intercept** | **95% CI** | | | **t** | **p** |
| --- | --- | --- | --- | --- | --- | --- |
| White Blood Cells | -2,473 | -7.55 | - | 2.6 | -0.955 | 0.372 |
| Lymphocytes | -2,707 | -5.95 | - | 0.54 | -1,634 | 0.141 |
| Procalcitonin | 6,372 | -0.76 | - | 13.5 | 1,752 | 0.130 |
| Lactate dehydrogenase | -3,717 | -9.56 | - | 2.13 | -1,247 | 0.259 |
| Interleukin 6 | 4,415 | -0.31 | - | 9.14 | 1,833 | 0.092 |
| D-dimer | 0.529 | -2.41 | - | 3.47 | 0.352 | 0.735 |

**S2B Table. Assessment of publication bias using Egger’s test on the meta-analyses including 10 or more studies on association between laboratory parameters and severity in patients with COVID-19**

| **Parameter** | **intercept** | **95% CI** | | | **t** | **p** |
| --- | --- | --- | --- | --- | --- | --- |
| White Blood Cells | -1.56 | -5.59 | - | 2.47 | -0.759 | 0.460 |
| Lymphocytes | 2,501 | -1.81 | - | 6.81 | 1,138 | 0.271 |
| Neutrophils | 0.34 | -6.89 | - | 7.57 | 0.092 | 0.928 |
| Platelets | 1,056 | -2.63 | - | 4.74 | 0.561 | 0.588 |
| C-reactive protein | -1,141 | -5.15 | - | 2.87 | -0.558 | 0.587 |
| Procalcitonin | 4,044 | -4.97 | - | 13.06 | 0.879 | 0.402 |
| Lactate dehydrogenase | -0.948 | -4.71 | - | 2.81 | -0.494 | 0.635 |
| Interleukin 6 | 8,072 | 5.19 | - | 10.96 | 5,482 | <0.001 |
| Creatinine | -1,781 | -4.11 | - | 0.54 | -1,502 | 0.171 |
