# Supplementary material for: Biomarkers of cytokine storm as red flags for severe and fatal COVID-19 cases: A living systematic review and meta-analysis
Source: PLoS One. 2021 Jun 29;16(6):e0253894. doi: 10.1371/journal.pone.0253894 (PMC8241122; doi:10.1371/journal.pone.0253894)
Supplement: S3 Table — (DOCX) [file pone.0253894.s004.docx]

**S3 Table. Excluded studies**

| **Study ID** | **Reason for exclusion** |
| --- | --- |
| Zhang M, 2020 (1) | It did not report eligible laboratory data |
| Xu Y-H, 2020 (2) | It did not report eligible laboratory data |
| Xu X, 2020 (3) | It did not report eligible laboratory data |
| Liu Y-C, 2020 (4) | Letter |
| Kong IP, 2020 (5) | It did not report eligible laboratory data |
| Song F, 2020 (6) | It did not report eligible laboratory data |
| Qu R, 2020 (7) | It did not report eligible laboratory data |
| Yin S, 2020 (8) | It did not report eligible laboratory data |
| Kim ES, 2020 (9) | It did not report eligible laboratory data |
| Li Y-K, 2020 (10) | It did not report eligible laboratory data |
| Lo IL, 2020 (11) | It did not report eligible laboratory data |
| Lescure F-X, 2020 (12) | It did not report eligible laboratory data |
| Li CX, 2020 (13) | It did not report eligible laboratory data |
| Lian J, 2020 (14) | It did not report eligible laboratory data |
| Zhong Q, 2020 (15) | It did not report eligible laboratory data |
| Wan S, 2020 (16) | It did not report eligible laboratory data |
| Liu K, 2020 (17) | It did not report eligible laboratory data |
| Liu M, 2020 (18) | It did not report eligible laboratory data |
| Xiong Y, 2020 (19) | It did not report eligible laboratory data |
| Young BE, 2020 (20) | It did not report eligible laboratory data |
| Li K, 2020 (21) | It did not report eligible laboratory data |
| Shi H, 2020 (22) | It did not report eligible laboratory data |
| Liu Y, 2020 (23) | It did not report eligible laboratory data |
| Chen N, 2020 (24) | It did not report severe versus non-severe patients |
| Sun Q, 2020 (25) | Letter |
| Xu Z, 2020 (26) | Case report |
| Wang D, 2020 (27) | It did not report eligible laboratory data |
| Chen W, 2020 (28) | Letter |
| Tang N, 2020 (29) | It did not report eligible laboratory data |
| Zhu W, 2020 (30) | It did not report severe versus non-severe patients |
| Yang X, 2020 (31) | It did not report eligible laboratory data |
| Huang Y, 2020 (32) | It did not report severe versus non-severe patients |
| Yang W, 2020 (33) | It did not report eligible laboratory data |
| Zhao X, 2020 (34) | It did not report eligible laboratory data |
| Ye G, 2020 (35) | It did not report eligible laboratory data |
| Feng Y, 2020 (36) | It did not report eligible laboratory data |
| Yang Y, 2020 (37) | It did not report eligible laboratory data |
| Magro G, 2020 (38) | Review |
| Han H, 2020 (39) | It did not report eligible laboratory data |
| Hong KS, 2020 (40) | It did not report eligible laboratory data |
| Ruan Q, 2020 (41) | It did not report eligible laboratory data |
| Huang C, 2020 (42) | It did not report eligible laboratory data |
| Sun Y, 2020 (43) | Ineligible population |
| Wang W, 2020 (44) | Different definition of severity groups |
| Gómez-Rial J, 2020 (45) | Incomplete laboratory data |
| Wendel Garcia PD, 2020 (46) | Ineligible population |
| Jain A, 2020 (47) | It did not report eligible laboratory data |
| Yang P-H, 2020 (48) | Different definition of severity groups |
| Popov GT, 2020 (49) | It did not report eligible laboratory data |
| Ren C, 2020 (50) | Different definition of severity groups |
| Liu D, 2020 (51) | Ineligible population |
| Sharma S, 2020 (52) | Ineligible population |
| Mughal M, 2020 (53) | Incomplete laboratory data |
| Chali TJ, 2020 (54) | Ineligible population |
| Wang D, 2020 (55) | It did not report eligible laboratory data |
| Jiang H, 2020 (56) | It did not report eligible laboratory data |
| Liu X, 2020 (57) | It did not report eligible laboratory data |
| Weng Z, 2020 (58) | It did not report eligible laboratory data |
| Maguire D, 2020 (59) | It did not report eligible laboratory data |
| Fu J, 2020 (60) | It did not report eligible laboratory data |
| Jin X, 2020 (61) | It did not report eligible laboratory data |
| Hao B, 2020 (62) | It did not report eligible laboratory data |
| Varim C, 2020 (63) | It did not report eligible laboratory data |
| Bi X, 2020 (64) | It did not report eligible laboratory data |
| Hou W, 2020 (65) | It did not report eligible laboratory data |
| Chen R, 2020 (66) | It did not report eligible laboratory data |
| Cao Z, 2020 (67) | It did not report eligible laboratory data |
| Cheng S, 2020 (68) | It did not report eligible laboratory data |
| Bastug A, 2020 (69) | It did not report eligible laboratory data |
| Rodríguez-Molinero A, 2020 (70) | It did not report eligible laboratory data |
| Miatech JL, 2020 (71) | It did not report eligible laboratory data |
| Sepulchre E, 2020 (72) | It did not report eligible laboratory data |
| Asghar MS, 2020 (73) | It did not report eligible laboratory data |
| Rokni M, 2020 (74) | It did not report eligible laboratory data |
| Petrak RM, 2020 (75) | Ineligible population |
| Dahan S, 2020 (76) | It did not report eligible laboratory data |
| Al Mutair A, 2020 (77) | Ineligible population |
| Xue G, 2020 (78) | It did not report eligible laboratory data |
| Ayaz A, 2020 (79) | It did not report eligible laboratory data |
| Awano N, 2020 (80) | It did not report eligible laboratory data |
| Huang H, 2020 (81) | It did not report eligible laboratory data |
| Chen Z, 2020 (82) | It did not report eligible laboratory data |
| Liu J, 2020 (83) | It did not report eligible laboratory data |
| Asghar MS, 2020 (84) | It did not report eligible laboratory data |
| Garcia Blasco, LM, 2020 (85) | It did not report eligible laboratory data |
| Zhang W, 2020 (86) | It did not report eligible laboratory data |
| Shi, Y, 2020 (87) | It did not report eligible laboratory data |
| Martos Pérez F, 2020 (88) | It did not report eligible laboratory data |
| Cai Q, 2020 (89) | Different definition of severity groups |
| Wu C, 2020 (90) | Different definition of severity groups |
| Dreher M, 2020 (91) | Different definition of severity groups |
| Guervilly C, 2020 (92) | Different definition of severity groups |
| Herold T, 2020 (93) | Different definition of severity groups |
| Tincati C, 2020 (94) | Different definition of severity groups |
| Maeda T, 2020 (95) | Different definition of severity groups |
| Jurado A, 2020 (96) | Different definition of severity groups |

**References**

1. Zhang MQ, Wang XH, Chen YL, Zhao KL, Cai YQ, An CL, et al. [Clinical features of 2019 novel coronavirus pneumonia in the early stage from a fever clinic in Beijing]. *Zhonghua Jie He He Hu Xi Za Zhi* 2020;43(3):215-8.

2. Xu Y-H, Dong J-H, An W-M, Lv X-Y, Yin X-P, Zhang J-Z, et al. Clinical and computed tomographic imaging features of novel coronavirus pneumonia caused by SARS-CoV-2. *Journal of Infection* 2020;80(4):394-400.

3. Xu X, Yu C, Qu J, Zhang L, Jiang S, Huang D, et al. Imaging and clinical features of patients with 2019 novel coronavirus SARS-CoV-2. *Eur J Nucl Med Mol Imaging* 2020;47(5):1275-80.

4. Liu Y-C, Liao C-H, Chang C-F, Chou C-C, Lin Y-R. A Locally Transmitted Case of SARS-CoV-2 Infection in Taiwan. *NEW ENGL J MED* 2020;382(11):1070-2.

5. Kong IP, Y.; Woo, Y.; Lee, J.; Cha, J.; Choi, J.; et al. Early Epidemiological and Clinical Characteristics of 28 Cases of Coronavirus Disease in South Korea. *Osong Public Health Res Perspect* 2020;11(1):8-14.

6. Song F, Shi N, Shan F, Zhang Z, Shen J, Lu H, et al. Emerging 2019 Novel Coronavirus (2019-nCoV) Pneumonia. *Radiology* 2020;295(1):210-7.

7. Qu R, Ling Y, Zhang YH, Wei LY, Chen X, Li XM, et al. Platelet-to-lymphocyte ratio is associated with prognosis in patients with coronavirus disease-19. *J Med Virol* 2020;92(9):1533-41.

8. Yin S, Huang M, Li D, Tang N. Difference of coagulation features between severe pneumonia induced by SARS-CoV2 and non-SARS-CoV2. *J Thromb Thrombolysis* 2020:1-4.

9. Kim ES, Chin BS, Kang CK, Kim NJ, Kang YM, Choi JP, et al. Clinical Course and Outcomes of Patients with Severe Acute Respiratory Syndrome Coronavirus 2 Infection: a Preliminary Report of the First 28 Patients from the Korean Cohort Study on COVID-19. *J Korean Med Sci* 2020;35(13):e142.

10. Li Y-K, Peng S, Li L-Q, Wang Q, Ping W, Zhang N, et al. Clinical and Transmission Characteristics of Covid-19 - A Retrospective Study of 25 Cases from a Single Thoracic Surgery Department. *Curr Med Sci* 2020;40(2):295-300.

11. Lo IL, Lio CF, Cheong HH, Lei CI, Cheong TH, Zhong X, et al. Evaluation of SARS-CoV-2 RNA shedding in clinical specimens and clinical characteristics of 10 patients with COVID-19 in Macau. *Int J Biol Sci* 2020;16(10):1698-707.

12. Lescure F-X, Bouadma L, Nguyen D, Parisey M, Wicky P-H, Behillil S, et al. Clinical and virological data of the first cases of COVID-19 in Europe: a case series. *The Lancet Infectious Diseases* 2020;20(6):697-706.

13. Li CX, Wu B, Luo F, Zhang N. [Clinical Study and CT Findings of a Familial Cluster of Pneumonia with Coronavirus Disease 2019 (COVID-19)]. *Sichuan Da Xue Xue Bao Yi Xue Ban* 2020;51(2):155-8.

14. Lian J, Jin X, Hao S, Cai H, Zhang S, Zheng L, et al. Analysis of Epidemiological and Clinical Features in Older Patients With Coronavirus Disease 2019 (COVID-19) Outside Wuhan. *Clin Infect Dis* 2020;71(15):740-7.

15. Zhong Q, Li Z, Shen X, Xu K, Shen Y, Fang Q, et al. [CT imaging features of patients with different clinical types of coronavirus disease 2019 (COVID-19)]. *Zhejiang Da Xue Xue Bao Yi Xue Ban* 2020;49(1):198-202.

16. Wan S, Xiang Y, Fang W, Zheng Y, Li B, Hu Y, et al. Clinical features and treatment of COVID-19 patients in northeast Chongqing. *J Med Virol* 2020;92(7):797-806.

17. Liu K, Chen Y, Lin R, Han K. Clinical features of COVID-19 in elderly patients: A comparison with young and middle-aged patients. *J Infect* 2020;80(6):e14-e8.

18. Liu M, He P, Liu HG, Wang XJ, Li FJ, Chen S, et al. [Clinical characteristics of 30 medical workers infected with new coronavirus pneumonia]. *Zhonghua Jie He He Hu Xi Za Zhi* 2020;43(0):E016.

19. Xiong Y, Sun D, Liu Y, Fan Y, Zhao L, Li X, et al. Clinical and High-Resolution CT Features of the COVID-19 Infection: Comparison of the Initial and Follow-up Changes. *Invest Radiol* 2020;55(6):332-9.

20. Young BE, Ong SWX, Kalimuddin S, Low JG, Tan SY, Loh J, et al. Epidemiologic Features and Clinical Course of Patients Infected With SARS-CoV-2 in Singapore. *JAMA* 2020;323(15):1488-94.

21. Li K, Wu J, Wu F, Guo D, Chen L, Fang Z, et al. The Clinical and Chest CT Features Associated With Severe and Critical COVID-19 Pneumonia. *Invest Radiol* 2020;55(6):327-31.

22. Shi H, Han X, Jiang N, Cao Y, Alwalid O, Gu J, et al. Radiological findings from 81 patients with COVID-19 pneumonia in Wuhan, China: a descriptive study. *The Lancet Infectious Diseases* 2020;20(4):425-34.

23. Liu Y, Yang Y, Zhang C, Huang F, Wang F, Yuan J, et al. Clinical and biochemical indexes from 2019-nCoV infected patients linked to viral loads and lung injury. *Science China Life Sciences* 2020;63(3):364-74.

24. Chen N, Zhou M, Dong X, Qu J, Gong F, Han Y, et al. Epidemiological and clinical characteristics of 99 cases of 2019 novel coronavirus pneumonia in Wuhan, China: a descriptive study. *The Lancet* 2020;395(10223):507-13.

25. Sun Q, Qiu H, Huang M, Yang Y. Lower mortality of COVID-19 by early recognition and intervention: experience from Jiangsu Province. *Annals of Intensive Care* 2020;10(1):33.

26. Xu Z, Shi L, Wang Y, Zhang J, Huang L, Zhang C, et al. Pathological findings of COVID-19 associated with acute respiratory distress syndrome. *Lancet Respir Med* 2020;8(4):420-2.

27. Wang D, Hu B, Hu C, Zhu F, Liu X, Zhang J, et al. Clinical Characteristics of 138 Hospitalized Patients With 2019 Novel Coronavirus–Infected Pneumonia in Wuhan, China. *JAMA* 2020;323(11):1061-9.

28. Chen W, Lan Y, Yuan X, Deng X, Li Y, Cai X, et al. Detectable 2019-nCoV viral RNA in blood is a strong indicator for the further clinical severity. *Emerg Microbes Infect* 2020;9(1):469-73.

29. Tang N, Li D, Wang X, Sun Z. Abnormal coagulation parameters are associated with poor prognosis in patients with novel coronavirus pneumonia. *J Thromb Haemost* 2020;18(4):844-7.

30. Zhu W, Xie K, Lu H, Xu L, Zhou S, Fang S. Initial clinical features of suspected coronavirus disease 2019 in two emergency departments outside of Hubei, China. *J Med Virol* 2020;92(9):1525-32.

31. Yang X, Yu Y, Xu J, Shu H, Xia Ja, Liu H, et al. Clinical course and outcomes of critically ill patients with SARS-CoV-2 pneumonia in Wuhan, China: a single-centered, retrospective, observational study. *Lancet Respir Med* 2020;8(5):475-81.

32. Huang Y, Tu M, Wang S, Chen S, Zhou W, Chen D, et al. Clinical characteristics of laboratory confirmed positive cases of SARS-CoV-2 infection in Wuhan, China: A retrospective single center analysis. *Travel Med Infect Dis* 2020;36:101606-.

33. Yang W, Cao Q, Qin L, Wang X, Cheng Z, Pan A, et al. Clinical characteristics and imaging manifestations of the 2019 novel coronavirus disease (COVID-19):A multi-center study in Wenzhou city, Zhejiang, China. *J Infect* 2020;80(4):388-93.

34. Zhao X, Liu B, Yu Y, Wang X, Du Y, Gu J, et al. The characteristics and clinical value of chest CT images of novel coronavirus pneumonia. *Clin Radiol* 2020;75(5):335-40.

35. Ye G, Pan Z, Pan Y, Deng Q, Chen L, Li J, et al. Clinical characteristics of severe acute respiratory syndrome coronavirus 2 reactivation. *J Infect* 2020;80(5):e14-e7.

36. Feng Y, Ling Y, Bai T, Xie Y, Huang J, Li J, et al. COVID-19 with Different Severities: A Multicenter Study of Clinical Features. *Am J Respir Crit Care Med* 2020;201(11):1380-8.

37. Yang Y, Shen C, Li J, Yuan J, Wei J, Huang F, et al. Plasma IP-10 and MCP-3 levels are highly associated with disease severity and predict the progression of COVID-19. *J Allergy Clin Immunol* 2020;146(1):119-27.e4.

38. Magro G. SARS-CoV-2 and COVID-19: Is interleukin-6 (IL-6) the 'culprit lesion' of ARDS onset? What is there besides Tocilizumab? SGP130Fc. *Cytokine X* 2020;2(2):100029.

39. Han H, Ma Q, Li C, Liu R, Zhao L, Wang W, et al. Profiling serum cytokines in COVID-19 patients reveals IL-6 and IL-10 are disease severity predictors. *Emerg Microbes Infect* 2020;9(1):1123-30.

40. Hong KS, Lee KH, Chung JH, Shin KC, Choi EY, Jin HJ, et al. Clinical Features and Outcomes of 98 Patients Hospitalized with SARS-CoV-2 Infection in Daegu, South Korea: A Brief Descriptive Study. *Yonsei Med J* 2020;61(5):431-7.

41. Ruan Q, Yang K, Wang W, Jiang L, Song J. Clinical predictors of mortality due to COVID-19 based on an analysis of data of 150 patients from Wuhan, China. *Intensive Care Med* 2020;46(5):846-8.

42. Huang C, Wang Y, Li X, Ren L, Zhao J, Hu Y, et al. Clinical features of patients infected with 2019 novel coronavirus in Wuhan, China. *The Lancet* 2020;395(10223):497-506.

43. Sun Y, Dong Y, Wang L, Xie H, Li B, Chang C, et al. Characteristics and prognostic factors of disease severity in patients with COVID-19: The Beijing experience. *Journal of Autoimmunity* 2020;112:102473.

44. Wang W, Zhao Z, Liu X, Liu G, Xie D, Xu Z, et al. Clinical features and potential risk factors for discerning the critical cases and predicting the outcome of patients with COVID-19. *Journal of Clinical Laboratory Analysis* 2020;34(10):e23547.

45. Gómez-Rial J, Currás-Tuala MJ, Rivero-Calle I, Gómez-Carballa A, Cebey-López M, Rodríguez-Tenreiro C, et al. Increased Serum Levels of sCD14 and sCD163 Indicate a Preponderant Role for Monocytes in COVID-19 Immunopathology. *Front Immunol* 2020;11:560381.

46. Wendel Garcia PD, Fumeaux T, Guerci P, Heuberger DM, Montomoli J, Roche-Campo F, et al. Prognostic factors associated with mortality risk and disease progression in 639 critically ill patients with COVID-19 in Europe: Initial report of the international RISC-19-ICU prospective observational cohort. *EClinicalMedicine* 2020;25.

47. Jain A, Chaurasia R, Sengar NS, Singh M, Mahor S, Narain S. Analysis of vitamin D level among asymptomatic and critically ill COVID-19 patients and its correlation with inflammatory markers. *Sci Rep* 2020;10(1):20191.

48. Yang P-H, Ding Y-B, Xu Z, Pu R, Li P, Yan J, et al. Increased circulating level of interleukin-6 and CD8+ T cell exhaustion are associated with progression of COVID-19. *Infectious Diseases of Poverty* 2020;9(1):161.

49. Popov GT, Baymakova M, Vaseva V, Kundurzhiev T, Mutafchiyski V. Clinical Characteristics of Hospitalized Patients with COVID-19 in Sofia, Bulgaria. *Vector-Borne and Zoonotic Diseases* 2020;20(12):910-5.

50. Ren C, Yao R-Q, Ren D, Li J-X, Li Y, Liu X-Y, et al. The Clinical Features and Prognostic Assessment of SARS-CoV-2 Infection-Induced Sepsis Among COVID-19 Patients in Shenzhen, China. *Front Med* 2020;7:570853-.

51. Liu D, Li R, Yu R, Wang Y, Feng X, Yuan Y, et al. Alteration of serum markers in COVID-19 and implications on mortality. *Clinical and Translational Medicine* 2020;10(3):e119.

52. Sharma S, Keswani P, Bhargava A, Sharma R, Shekhawat A, Bhandari S. Overview of Early Cases of Coronavirus Disease 2019 (COVID-19) at a Tertiary Care Centre in North India. *Ann Acad Med Singap* 2020;49(7):449-55.

53. Mughal M, Kaur IP, Jaffery A, Dalmacion DL, Kramer V, Patton C, et al. CAN INFLAMMATORY MARKERS PREDICT THE SUCCESSFUL EXTUBATION IN PATIENTS WITH COVID-19? *CHEST* 2020;158(4):A600.

54. Chali TJ, ALjwaid HO, Kashan IS, Tarish HR, AlJanabi MH. The Role of IL6, IL8, TNF α, INF α and some of the positive acute-phase protein in the prognosis of SARS COVID-19. *The prognosis of SARS COVID-19* 2020;23(14).

55. Wang D, Li R, Wang J, Jiang Q, Gao C, Yang J, et al. Correlation analysis between disease severity and clinical and biochemical characteristics of 143 cases of COVID-19 in Wuhan, China: a descriptive study. *BMC Infectious Diseases* 2020;20.

56. Jiang H, Guo W, Shi Z, Jiang H, Zhang M, Wei L, et al. Clinical imaging characteristics of inpatients with coronavirus disease-2019 in Heilongjiang Province, China: a retrospective study. *Aging (Albany NY)* 2020;12(14):13860-8.

57. Liu X, Yue X, Liu F, Wei L, Chu Y, Bao H, et al. Analysis of clinical features and early warning signs in patients with severe COVID-19: A retrospective cohort study. *PLOS ONE* 2020;15(6):e0235459.

58. Weng Z, Chen Q, Li S, Li H, Zhang Q, Lu S, et al. ANDC: an early warning score to predict mortality risk for patients with Coronavirus Disease 2019. *Journal of Translational Medicine* 2020;18(1):328.

59. Maguire D, Woods M, Richards C, Dolan R, Wilson Veitch J, Sim W, et al. Prognostic factors in patients admitted to an urban teaching hospital with COVID-19 infection. *Journal of Translational Medicine* 2020;18.

60. Fu J, Kong J, Wang W, Wu M, Yao L, Wang Z, et al. The clinical implication of dynamic neutrophil to lymphocyte ratio and D-dimer in COVID-19: A retrospective study in Suzhou China. *Thromb Res* 2020;192:3-8.

61. Jin X, Lian JS, Hu JH, Gao J, Zheng L, Zhang YM, et al. Epidemiological, clinical and virological characteristics of 74 cases of coronavirus-infected disease 2019 (COVID-19) with gastrointestinal symptoms. *Gut* 2020;69(6):1002-9.

62. Hao B, Sotudian S, Wang T, Xu T, Hu Y, Gaitanidis A, et al. Early prediction of level-of-care requirements in patients with COVID-19. *Elife* 2020;9.

63. Varim C, Yaylaci S, Demirci T, Kaya T, Nalbant A, Dheir H, et al. Neutrophil count to albumin ratio as a new predictor of mortality in patients with COVID-19 1nfection. *Revista da Associação Médica Brasileira* 2020;66:77-81.

64. Bi X, Su Z, Yan H, Du J, Wang J, Chen L, et al. Prediction of severe illness due to COVID-19 based on an analysis of initial Fibrinogen to Albumin Ratio and Platelet count. *Platelets* 2020;31(5):674-9.

65. Hou W, Zhang W, Jin R, Liang L, Xu B, Hu Z. Risk factors for disease progression in hospitalized patients with COVID-19: a retrospective cohort study. *Infect Dis (Lond)* 2020;52(7):498-505.

66. Chen R, Liang W, Jiang M, Guan W, Zhan C, Wang T, et al. Risk Factors of Fatal Outcome in Hospitalized Subjects With Coronavirus Disease 2019&#xa0;From a Nationwide Analysis in China. *CHEST* 2020;158(1):97-105.

67. Cao Z, Li T, Liang L, Wang H, Wei F, Meng S, et al. Clinical characteristics of Coronavirus Disease 2019 patients in Beijing, China. *PloS one* 2020;15(6):e0234764-e.

68. Cheng S, Wu D, Li J, Zou Y, Wan Y, Shen L, et al. Risk factors for the critical illness in SARS-CoV-2 infection: a multicenter retrospective cohort study. *Respir Res* 2020;21(1):277.

69. Bastug A, Bodur H, Erdogan S, Gokcinar D, Kazancioglu S, Kosovali BD, et al. Clinical and laboratory features of COVID-19: Predictors of severe prognosis. *Int Immunopharmacol* 2020;88:106950.

70. Rodríguez-Molinero A, Gálvez-Barrón C, Miñarro A, Macho O, López GF, Robles MT, et al. Association between COVID-19 prognosis and disease presentation, comorbidities and chronic treatment of hospitalized patients. *PLOS ONE* 2020;15(10):e0239571.

71. Miatech JL, Yaslik CP, Tarleton HE, West D, Kellum W, McKnight M, et al. Retrospective Analysis of Inflammatory Markers and Patient Characteristics in Hospitalized Covid-19 Patients: An Early Experience in Louisiana. *Cureus* 2020;12(9):e10257-e.

72. Sepulchre E, Pittie G, Stojkovic V, Haesbroek G, Crama Y, Schyns M, et al. Covid-19: contribution of clinical characteristics and laboratory features for early detection of patients with high risk of severe evolution. *Acta Clin Belg* 2020:1-7.

73. Asghar MS, Haider Kazmi SJ, Khan NA, Akram M, Jawed R, Rafaey W, et al. Role of Biochemical Markers in Invasive Ventilation of Coronavirus Disease 2019 Patients: Multinomial Regression and Survival Analysis. *Cureus* 2020;12(8):e10054.

74. Rokni M, Ahmadikia K, Asghari S, Mashaei S, Hassanali F. Comparison of clinical, para-clinical and laboratory findings in survived and deceased patients with COVID-19: diagnostic role of inflammatory indications in determining the severity of illness. *BMC Infectious Diseases* 2020;20(1):869.

75. Petrak RM, Skorodin NC, Van Hise NW, Fliegelman RM, Pinsky J, Didwania V, et al. Tocilizumab as a Therapeutic Agent for Critically Ill Patients Infected with SARS-CoV-2. *Clin Transl Sci* 2020.

76. Dahan S, Segal G, Katz I, Hellou T, Tietel M, Bryk G, et al. Ferritin as a Marker of Severity in COVID-19 Patients: A Fatal Correlation. *Isr Med Assoc J* 2020;22(8):494-500.

77. Al Mutair A, Alhumaid S, Alhuqbani WN, Zaidi ARZ, Alkoraisi S, Al-Subaie MF, et al. Clinical, epidemiological, and laboratory characteristics of mild-to-moderate COVID-19 patients in Saudi Arabia: an observational cohort study. *European Journal of Medical Research* 2020;25(1):61.

78. Xue G, Gan X, Wu Z, Xie D, Xiong Y, Hua L, et al. Novel serological biomarkers for inflammation in predicting disease severity in patients with COVID-19. *Int Immunopharmacol* 2020;89(Pt A):107065.

79. Ayaz A, Arshad A, Malik H, Ali H, Hussain E, Jamil B. Risk factors for intensive care unit admission and mortality in hospitalized COVID-19 patients. *Acute Crit Care* 2020;35(4):249-54.

80. Awano N, Inomata M, Kuse N, Tone M, Takada K, Muto Y, et al. Serum KL-6 level is a useful biomarker for evaluating the severity of coronavirus disease 2019. *Respir Investig* 2020;58(6):440-7.

81. Huang H, Cai S, Li Y, Li Y, Fan Y, Li L, et al. Prognostic Factors for COVID-19 Pneumonia Progression to Severe Symptoms Based on Earlier Clinical Features: A Retrospective Analysis. *Front Med (Lausanne)* 2020;7:557453.

82. Chen Z, Zhang F, Hu W, Chen Q, Li C, Wu L, et al. Laboratory markers associated with COVID-19 progression in patients with or without comorbidity: A retrospective study. *J Clin Lab Anal* 2021;35(1):e23644.

83. Liu J, Liu Z, Jiang W, Wang J, Zhu M, Song J, et al. Clinical predictors of COVID-19 disease progression and death: Analysis of 214 hospitalised patients from Wuhan, China. *Clin Respir J* 2020.

84. Asghar MS, Khan NA, Haider Kazmi SJ, Ahmed A, Hassan M, Jawed R, et al. Hematological parameters predicting severity and mortality in COVID-19 patients of Pakistan: a retrospective comparative analysis. *J Community Hosp Intern Med Perspect* 2020;10(6):514-20.

85. Garcia Blasco LM, Alfaro Martínez JJ, Quílez Toboso R, Sánchez Sáez P, Rodríguez Marín Y, García Aragonés L, et al. Albumin levels and its association with outcomes in a Serie of Covid-19 patients in Spain. *Clinical Nutrition ESPEN* 2020;40:621.

86. Zhang W, Zhang Z, Ye Y, Luo Y, Pan S, Qi H, et al. Lymphocyte percentage and hemoglobin as a joint parameter for the prediction of severe and nonsevere COVID-19: a preliminary study. *Ann Transl Med* 2020;8(19):1231.

87. Shi Y OJ, Chen X, Tan M, Li F, Liu Y. Expressions of multiple inflammation markers in the patients with COVID-19 and their clinical values. *Chinese Journal of Laboratory Medicine* 2020;43(4).

88. Martos Pérez F, Luque del Pino J, Jiménez García N, Mora Ruiz E, Asencio Méndez C, García Jiménez JM, et al. Comorbidity and prognostic factors on admission in a COVID-19 cohort of a general hospital. *Rev Clin Esp (Barc)* 2020.

89. Cai Q, Huang D, Ou P, Yu H, Zhu Z, Xia Z, et al. COVID-19 in a designated infectious diseases hospital outside Hubei Province, China. *Allergy* 2020.

90. Wu C, Chen X, Cai Y, Xia J, Zhou X, Xu S, et al. Risk Factors Associated With Acute Respiratory Distress Syndrome and Death in Patients With Coronavirus Disease 2019 Pneumonia in Wuhan, China. *JAMA Intern Med* 2020.

91. Dreher M, Kersten A, Bickenbach J, Balfanz P, Hartmann B, Cornelissen C, et al. The Characteristics of 50 Hospitalized COVID-19 Patients With and Without ARDS. *Dtsch Arztebl Int* 2020;117(16):271-8.

92. C G, S B, F S, R C, G L, E A, et al. Circulating Endothelial Cells as a Marker of Endothelial Injury in Severe COVID -19. *The Journal of infectious diseases* 2020;222(11):1789-93.

93. Herold T, Jurinovic V, Arnreich C, Lipworth BJ, Hellmuth JC, Bergwelt-Baildon MV, et al. Elevated levels of IL-6 and CRP predict the need for mechanical ventilation in COVID-19. *J Allergy Clin Immunol* 2020.

94. C T, ES C, M G, R B, A dAM, G M. Heightened Circulating Interferon-Inducible Chemokines, and Activated Pro-Cytolytic Th1-Cell Phenotype Features Covid-19 Aggravation in the Second Week of Illness. *Frontiers in immunology* 2020;11:580987.

95. Maeda T, Obata R, Rizk DD, Kuno T. The association of interleukin-6 value, interleukin inhibitors, and outcomes of patients with COVID-19 in New York City. *J Med Virol* 2021;93(1):463-71.

96. Jurado A, Martín MC, Abad-Molina C, Orduña A, Martínez A, Ocaña E, et al. COVID-19: age, Interleukin-6, C-reactive protein, and lymphocytes as key clues from a multicentre retrospective study. *Immun Ageing* 2020;17:22.
